# Network geometry, topology, and spectral analysis in global stock markets: Insights from using the Ricci curvature, Euler characteristic, and random matrix theory

**DOI:** 10.1371/journal.pone.0347767

**Published:** 2026-05-12

**Authors:** Andy Domínguez-Monterroza, Antonio Jiménez-Martín, Alfonso Mateos Caballero

**Affiliations:** 1 Departamento de Inteligencia Artificial, Universidad Politécnica de Madrid, Madrid, Spain; 2 Departamento de Matemáticas, Pontificia Universidad Javeriana, Bogotá, Colombia; University of Minnesota, UNITED STATES OF AMERICA

## Abstract

The global stock market network exhibits complex patterns of interdependence that are central to systemic stability. This study proposes a multi-perspective framework integrating random matrix theory, Ricci curvature, and the Euler characteristic to characterize synchronization, geometric robustness, and global structural organization in financial networks. The maximum eigenvalue captures collective market behavior, Ricci curvature quantifies local fragility, and the Euler characteristic summarizes global topological cohesion.The framework is further complemented by entropy-based descriptors that capture spectral concentration and geometric heterogeneity, thereby provideng complementary multiscale descriptors to characterize of systemic fragility. Using daily data from major global stock indices (2017–2022), we identify pronounced structural transitions during the COVID-19 pandemic. The dynamic analysis also reveals a secondary structural reconfiguration in early 2022, temporally aligned with the Russia–Ukraine shock, suggesting that geometric and topological descriptors are sensitive to heterogeneous stress events. The results demonstrate that integrating spectral, geometric, and topological perspectives provides complementary insights into market resilience and crisis-driven structural change.

## 1 Introduction

The global stock market network constitutes a complex and dynamic system, wherein financial indices from diverse economies interact to reflect both localized market dynamics and broader macroeconomic forces, which are essential for detecting structural trends [1–3]. A comprehensive understanding of network structural properties is therefore central to identifying stability patterns, assessing systemic risk, and evaluating resilience, particularly in the presence of large-scale economic disruptions [4–6]. In this context, network science has emerged as a rigorous analytical framework, providing tools to quantify interdependencies, characterize structural evolution, and model systemic responses to exogenous shocks across interconnected financial markets [7,8].

Traditional network analysis in finance has predominantly focused on metrics such as degree centrality, betweenness, and community structure. While informative, these descriptors primarily capture node-level or local connectivity properties and offer limited access to geometric and spectral characteristics that govern collective and global network behavior. *Random matrix theory* (RMT) provides a complementary perspective by examining the spectral properties of correlation matrices, with particular emphasis on the maximum eigenvalue, which serves as a robust indicator of market synchronization and systemic risk [9,10]. Elevated eigenvalues are commonly associated with heightened collective behavior, a hallmark of financial crises during which global markets exhibit increased synchronization [10,11]. Integrating RMT with network-based methodologies thus enables a more comprehensive characterization of structural shifts and market stability.

In addition to spectral analysis, recent developments in network geometry and topology offer additional insights into financial system resilience. The Ricci curvature, rooted in Riemannian geometry, quantifies the strength and robustness of connections within a network [12]. In financial networks, positive Ricci curvature indicates cohesive and resilient subnetworks, whereas negative curvature highlights bridge-like or fragile connections that may represent systemic vulnerabilities [13,14]. This geometric descriptor has been increasingly applied to complex systems, including financial markets, as a means of assessing structural robustness and potential pathways for shock propagation [7,15].

Complementarily, the Euler characteristic constitutes a global topological invariant that summarizes the overall structural complexity of a network. Although widely used in other scientific domains, its application to financial network analysis remains comparatively limited. By encoding the balance between nodes and edges, the Euler characteristic provides information on fragmentation, cohesion, and global integration across different temporal and economic regimes [7,16]. When combined with Ricci curvature, it supports a joint geometric–topological perspective that can reflect both local robustness and global structural organization, complementing traditional metrics in the characterization of systemic fragility.

The COVID-19 crisis intensified interest in dynamic representations of market interconnectedness, with empirical studies documenting abrupt increases in synchronization and contagion across global equity markets during periods of acute stress [17–19]. However, much of this evidence remains dispersed across distinct methodological traditions, which typically treat spectral, geometric, and topological descriptors as separate analytical lenses rather than as components of a unified characterization of systemic fragility.

Motivated by theoretical links between curvature, entropy, and robustness [13], an additional open question concerns whether information-theoretic descriptors co-evolve with geometric and spectral indicators of stress. In particular, spectral entropy derived from eigenvalue distributions can quantify reductions in complexity associated with market synchronization, while curvature-based (geometric) network entropy can summarize heterogeneity in edge-level transport and deformation patterns. Jointly tracking these entropy measures alongside Ricci curvature and RMT indicators may therefore enhance the interpretation of systemic fragility across multiple scales.

This study proposes a multi-perspective framework for analyzing the structural properties of global stock market networks by integrating RMT, Ricci curvature, and the Euler characteristic. The maximum RMT eigenvalue captures periods of extreme market synchronization, Ricci curvature provides a localized assessment of resilience and fragility at the edge level, and the Euler characteristic reflects global topological cohesion. Together, these descriptors provide an integrated description of the market structure that goes beyond standard network summaries.

The primary objective of this study is methodological: to develop and empirically evaluate a unified analytical pipeline that integrates spectral, geometric, and topological measures, augmented with entropy-based descriptors, in order to obtain a multiscale representation of systemic fragility in global stock market networks.

Focusing on the period from 2017 to 2022, this study addresses the following research questions:

- How do Ricci curvature, the Euler characteristic, and the maximum eigenvalue vary across periods of market stability and instability?
- What insights do these measures provide regarding the cohesion and robustness of global market interconnections?
- How do these descriptors dynamically evolve in response to external shocks, particularly during the COVID-19 pandemic?
- How do entropy-based measures co-evolve with curvature and spectral indicators, and to what extent do they reveal complementary information about systemic fragility?
- To what extent does the integrated framework detect structural changes that individual metrics may fail to capture when considered in isolation?

Methodologically, the study constructs annual and sliding-window correlation networks from daily log returns of major global stock indices. Ollivier–Ricci curvature is employed to characterize local connectivity patterns, the Euler characteristic is used to assess global topological structure, and RMT is applied to analyze spectral properties, with the maximum eigenvalue serving as a proxy for collective market behavior.

To situate the contribution within contemporary research, Section 2 reviews recent developments in spectral (RMT), geometric (Ricci curvature), and topological approaches to financial networks, including persistent homology as a general topological data analysis framework. In this study, however, the topological perspective is operationalized through the Euler characteristic as a low-order global invariant, providing an interpretable summary of network cohesion and fragmentation at the system level.

## 2 Related works

The analysis of financial networks using advanced mathematical frameworks has expanded substantially in recent years, particularly following major economic disruptions such as the COVID-19 pandemic and subsequent geopolitical events. This section reviews recent developments across three interconnected methodological domains: spectral analysis based on random matrix theory, geometric approaches employing Ricci curvature, and topological methods grounded in persistent homology. Rather than providing a purely descriptive survey, the focus is placed on how each methodological stream captures complementary yet incomplete aspects of systemic risk, thereby motivating the need for integrated multiscale frameworks.

### 2.1 Spectral methods and random matrix theory

Random matrix theory (RMT) has become a foundational tool for characterizing correlation structures in financial markets. Seminal contributions by Laloux et al. [9] and Plerou et al. [10] demonstrated that the eigenvalue spectrum of empirical correlation matrices largely follows the predictions of the Marchenko–Pastur distribution, with deviations corresponding to economically meaningful information rather than noise. These results established the interpretation of the largest eigenvalue as a proxy for market-wide synchronization.

Subsequent studies have extended this framework to a variety of market settings. Tang et al. [20] analyzed Chinese and U.S. equity markets, showing that while most eigenvalues remain within theoretical bounds, the leading eigenvalue consistently reflects crisis-induced synchronization. Molero-González et al. [21] further demonstrated that the dominant eigenvalue captures spillover effects across developed and emerging markets, while subleading eigenvalues encode sectoral and countercyclical dynamics.

Despite their empirical relevance, spectral approaches remain inherently global in nature. They quantify the concentration of variance and collective behavior but do not provide explicit information about the underlying network geometry or the localization of fragility at the level of individual market connections. In particular, increases in the largest eigenvalue do not distinguish between diffuse correlation growth and structurally critical transmission pathways.

Recent methodological refinements have addressed limitations of classical RMT assumptions. Montiel Olea et al. [22] proposed hierarchical extensions of the Marchenko–Pastur framework to accommodate post-2020 deviations and multiple characteristic timescales. While these advances improve spectral modeling accuracy, they remain confined to eigenvalue statistics and do not incorporate explicit network representations or edge-level robustness measures.

### 2.2 Geometric approaches: Ricci curvature in financial networks

The application of differential geometry to financial networks represents a comparatively recent yet rapidly expanding research direction. Ollivier–Ricci curvature [12] provides a principled measure of network robustness by quantifying how probability mass is transported between neighboring nodes. Positive curvature is associated with cohesive and resilient subnetworks, whereas negative curvature highlights bridge-like connections that may correspond to structural vulnerabilities.

Sandhu et al. [13] were among the first to apply Ricci curvature to financial markets, showing that curvature exhibits characteristic changes during periods of instability and crisis. Subsequent studies have confirmed its empirical relevance across markets and time horizons [7,23]. In particular, Samal et al. [7] demonstrated that multiple curvature variants capture bubbles and crashes with greater sensitivity than several classical volatility-based indicators.

However, curvature-based approaches remain fundamentally local. They characterize robustness at the edge or neighborhood level but do not directly summarize the global organization of the financial system or its spectral synchronization properties. Moreover, curvature alone cannot distinguish whether market-wide transitions arise from homogeneous weakening across the network or from stress concentration along a limited set of critical connections.

Recent efforts have explored partial integrations. Kulkarni et al. [24] combined Ricci curvature with topological data analysis in the context of the Indian stock market, showing that multi-method approaches can yield richer insights than single-metric analyses. Nevertheless, such integrations remain partial and lack a unified framework that systematically combines spectral, geometric, and topological descriptors.

### 2.3 Topological methods: Persistent homology and financial time series

*Topological data analysis* (TDA), particularly persistent homology, has emerged as a powerful methodology for detecting regime shifts and critical transitions in financial systems. Gidea and Katz [25] showed that persistence landscapes exhibit characteristic growth patterns prior to major financial crashes, highlighting the potential of topological features as early-warning indicators.

Subsequent work provided both theoretical and empirical support for these findings. Akingbade et al. [26] established a theoretical connection between TDA-based indicators and *log-periodic power law singularity* (LPPLS) dynamics, while Guo et al. [27] demonstrated that persistence-based metrics capture abrupt correlation regime shifts during major public events.

Recent methodological advances have further extended the applicability of TDA. De Jesus et al. [28] showed that topological features improve univariate time-series forecasting, and Arvanitis and Detsis [29] applied persistent homology to cryptocurrency markets. Despite preserving multiscale structural information, TDA-based approaches are typically decoupled from explicit network representations and do not incorporate economic notions of edge robustness or spectral synchronization. As a result, persistent homology alone provides limited interpretability regarding how specific market connections contribute to systemic fragility.

### 2.4 Integration and systemic risk assessment

An increasing body of research recognizes that systemic risk emerges from the interaction of multiple structural mechanisms. Sun [30] showed that risk-transmission patterns vary substantially across institution types, underscoring the heterogeneity of shock-propagation mechanisms. Chen et al. [31] demonstrated that systemic risk arises from the interplay between network structure and market dynamics rather than from either component in isolation.

Nevertheless, most existing studies adopt one methodological perspective at a time: (i) spectral approaches based on RMT, (ii) geometric approaches based on curvature, or (iii) topological approaches based on algebraic invariants or persistent homology. While a limited number of works explore partial combinations [7,24], there is still no unified analytical framework that systematically integrates spectral synchronization, local geometric fragility, and global topological cohesion within a single multiscale representation.

Moreover, although the impact of COVID-19 on financial networks has been extensively documented [17–19], most empirical studies focus on short-term correlation spikes or isolated classes of network metrics. The post-pandemic recovery phase and subsequent geopolitical shocks, such as the 2022 Russia–Ukraine conflict, remain comparatively underexplored from an integrated geometric–spectral–topological perspective.

In this context, the present study contributes to the literature by proposing a unified analytical pipeline that integrates random matrix theory, Ollivier–Ricci curvature, the Euler characteristic, and entropy-based descriptors within a sliding-window framework. This integration enables the simultaneous assessment of market-wide synchronization, local structural fragility, global topological cohesion, and informational disorder, thereby extending existing single-perspective approaches.

## 3 Methods

### 3.1 Data and log returns

This study employs the daily closing prices of major financial stock indices from global markets over the period 2017–2022, sourced from Yahoo Finance via its Python API. This timeframe was selected to capture a complete crisis cycle: a pre-crisis baseline (2017–2019), the COVID-19 shock (2020), and the recovery period (2021–2022). These indices serve as key benchmarks for their respective national stock markets, encapsulating overall economic performance and investor sentiment within each country.

Initially, 44 stock indices from economies across all continents were considered. To ensure data integrity and consistency, however, indices with more than 20% missing data were excluded, resulting in a final selection of 34 indices for analysis. These indices, identified by their corresponding Yahoo Finance tickers, encompass a broad spectrum of developed and emerging markets, as detailed in Table 1.

**Table 1:** List of Countries, Regions, and Index Symbol.

Country	Region	Index Symbol
Japan	Asia	N225
Hong Kong	Asia	HSI
China	Asia	000001.SS
Taiwan	Asia	TWII
India	Asia	BSESN
Singapore	Asia	STI
United Kingdom	Europe	FTSE
Switzerland	Europe	SSMI
Spain	Europe	IBEX
Germany	Europe	GDAXI
Finland	Europe	OMXH25
Portugal	Europe	PSI20.LS
France	Europe	FCHI
Italy	Europe	FTSEMIB.MI
Netherlands	Europe	AEX
Belgium	Europe	BFX
Norway	Europe	OSEAX
Sweden	Europe	OMXC25
Greece	Europe	ATX
United States	Americas	GSPC
Brazil	Americas	BVSP
Mexico	Americas	MXX
Argentina	Americas	MERV
Canada	Americas	GSPTSE
Chile	Americas	IPSA
Peru	Americas	SPBLPGPT
Australia	Oceania	AXJO
New Zealand	Oceania	NZ50
South Africa	Middle East & Africa	JTOPI
Egypt	Middle East & Africa	EGX30
Israel	Middle East & Africa	TA125.TA
Qatar	Middle East & Africa	QSI
UAE	Middle East & Africa	ADX
Saudi Arabia	Middle East & Africa	TASI

Let $P_{i}(t)$ denote the closing price of stock index *i* on day *t*. To normalize differences in price scales and maintain statis*t*ical robustness, the logarithmic returns are computed as:


$$r_{i}(t)=\log\left(\frac{P_{i}(t)}{P_{i}(t-1)}\right),$$ (1)

This transformation standardizes the data and mitigates distortions arising from variations in absolute price levels [32]. It is widely employed in financial time series due to its properties, which enhance statistical consistency and facilitate the modeling of market dynamics.

### 3.2 Correlation matrix and thresholding

The interdependencies between stock indices are quantified using the Pearson correlation coefficient $\rho_{ij}$, defined as:


$$\rho_{ij}=\frac{𝔼[(r_{i}-{\bar{r}}_{i})(r_{j}-{\bar{r}}_{j})]}{\sigma_{i}\sigma_{j}},$$ (2)

where 𝔼 denotes the expectation, ${\bar{r}}_{i}$ represents the mean return of index *i*, and $\sigma_{i}$ corresponds to its standard deviation. This computation yields an N×N correlation matrix for each year, where *N* denotes the number of stock indices analyzed.

To extract significant relationships, a threshold is applied based on the average correlation:


$$\langle \rho \rangle =\frac{2}{N(N-1)}\sum_{i<j}\rho_{ij}.$$ (3)

Edges are established between nodes *i* and *j* whenever the pairwise correlation satisfies $\rho_{ij}\geq \langle \rho \rangle$, where ⟨ρ⟩ denotes the average correlation within the corresponding window. This data-adaptive thresholding scheme allows network density to adjust endogenously to variations in overall market synchronization, reducing the risk of mechanically induced densification when correlation levels shift across market conditions, and improving temporal comparability across regimes [33–35].

### 3.3 Distance metric

To convert correlation values into distances while preserving the inverse relationship (i.e., higher correlation corresponds to shorter distance), the following metric is employed [32,36]:


$$D_{ij}=\sqrt{2(1-\rho_{ij})}.$$ (4)

This metric satisfies the ultrametric properties and is widely utilized in financial networks analysis. The criterion for edge inclusion in the graph is determined based on the average correlation threshold:


$$D_{\text{threshold}}=\sqrt{2(1-\langle \rho \rangle )}.$$ (5)

### 3.4 Network construction

The financial network is modeled as *G* = (*V*, *E*), where *V* represents the set of nodes (indices) and *E* denotes the set of edges. An edge (*i*, *j*) is established if:


$$D_{ij}\leq D_{\text{threshold}},$$ (6)

ensuring that only significant market relationships are incorporated into the network structure. The weight of each edge is assigned as $D_{ij}$ for subsequent use in curvature computations.

### 3.5 Ollivier-Ricci curvature

The Ollivier-Ricci curvature is employed to evaluate the network’s connectivity and structural cohesion. For each edge (*i*, *j*), the curvature κ(i,j) is defined as:


$$\kappa (i,j)=1-\frac{W_{1}(\mu_{i},\mu_{j})}{d(i,j)},$$ (7)

where $W_{1}(\mu_{i},\mu_{j})$ represents the Wasserstein-1 distance between the local probability distributions $\mu_{i}$ and $\mu_{j}$ associated with nodes *i* and *j*, while *d*(*i*, *j*) denotes the shortest-path distance between these nodes in the network [12]. In unweighted networks, *d*(*i*, *j*) is conventionally set to 1.

The average Ricci curvature of the network is computed by averaging κ(i,j) over all edges. A positive κ(i,j) value indicates strong connectivity and structural robustness, whereas a negative value suggests potential bottlenecks or weak links in the network [14]. Higher positive values correspond to clusters of densely interconnected nodes, signifying structurally stable regions. Conversely, a negative Ricci curvature indicates weaker more tenuous connections between nodes *i* and *j*, identifying a bottleneck or bridge in the network. These connections often link otherwise separate communities, highlighting areas of structural vulnerability or volatility areas in the financial system.

In essence, the Ricci curvature differentiates between stable clusters and critical links within the network. By analyzing its temporal evolution, it is possible to detect structural shifts in the global stock market, capturing changes in connectivity and resilience across different regions or sectors [15].

### 3.6 Euler characteristic

The Euler characteristic χ, a fundamental topological invariant, provides a global summary of the network’s structure and is defined as::


χ=|V|−|E|, (8)

where |*V*| and |*E*| represent the number of nodes and edges, respectively.

Positive values indicate fragmented networks, whereas negative values suggest a higher degree of connectivity [16,37]. As a measure of topological complexity, the Euler characteristic offers insight into the presence of cycles and the overall cohesion of the structure. Higher, more positive values of the Euler characteristic generally correspond to a sparser network with fewer connections, implying relative market fragmentation, where clusters of stock indices exhibit weak interdependencies [16].

In a financial context, an increase in the Euler characteristic could signal periods of lower correlation across indices, reflecting a greater degree of segmentation between regions or sectors. Conversely, lower, more negative Euler characteristic values indicate a denser network with a higher number of connections relative to nodes, suggesting stronger global integration [37]. This pattern is commonly observed during periods of heightened market interconnectedness, where stock indices exhibit stronger correlations across regions, often corresponding to increased financial interdependence or heightened volatility.

The Euler characteristic serves as a topological assessment of the network’s complexity, providing an overview of how tightly or loosely connected the global stock market is across different periods. Variations in the Euler characteristic provide a means to assess shifts in the degree of market integration and the overall structural stability of the financial network. By analyzing its temporal evolution, it is possible to identify periods of increased fragmentation or enhanced connectivity, offering insights into changes in global market cohesion.

### 3.7 Random matrix theory

RMT is employed to investigate the spectral properties of the correlation matrix. Eigenvalue analysis in financial correlation matrices has been extensively utilized to distinguish between stochastic fluctuations and meaningful market dynamics [38–40]. A key spectral measure used in this analysis is the maximum eigenvalue, $\lambda_{\max}$, which serves as an indicator of collective market behavior. The eigenvalues $\lambda_{i}$ of the correlation matrix are computed, and the largest eigenvalue is extracted:


$$\lambda_{\max}=\max(\lambda_{i}).$$ (9)

A higher $\lambda_{\max}$ reflects stronger market-wide correlations, while deviations from the Marchenko-Pastur distribution suggest the presence of non-random, structurally significant market patterns beyond stochastic noise [9,10,41].

### 3.8 Dynamic analysis of network metrics

To capture the temporal evolution of network properties, a sliding-window approach is implemented with a window length of τ=250 trading days (approximately one year) and a step size of Δτ=5 days. This design balances temporal resolution and statistical reliability, enabling a fine-grained yet stable characterization of structural variations in the financial network over time [7].

The choice of a one-year window is motivated by the need to suppress short-term market noise while preserving mesoscopic structural trends associated with regime transitions and systemic reorganization. Larger windows also improve the stability of correlation estimates and derived network descriptors, thereby enhancing the interpretability of long-term structural dynamics.

To assess the robustness of the sliding-window design and to address the trade-off between temporal resolution and statistical reliability, we performed a systematic sensitivity analysis over multiple window sizes τ∈{22,60,90,132,250} and step sizes Δτ∈{1,5,22}. This analysis evaluates the stability of the estimated network descriptors under alternative temporal aggregations and sampling granularities. The full sensitivity assessment is reported in the supplementary material (Figs. S1–S2 in S1 File), and informs the parameter choices adopted in the main dynamic analysis.

Based on these findings, τ=250 and Δτ=5 are adopted as a compromise between temporal smoothness and structural interpretability. This configuration provides a stable representation of long-term market regimes while retaining sufficient resolution to track major systemic transitions, including the COVID-19 shock.

For each sliding window [t,t+τ] with step size Δτ, the following computations are performed:

1. *Correlation matrix computation:* Pearson correlation coefficients $\rho_{ij}(t)$ are computed for stock indices within the window.
2. *Spectral analysis (RMT):* The maximum eigenvalue $\lambda_{\max}(t)$ of the correlation matrix is extracted as a measure of collective market synchronization.
3. *Thresholding and network construction:* A financial network is constructed by retaining edges (*i*,*j*) such that $\rho_{ij}(t)\geq \langle \rho_{ij}(t)\rangle_{\tau }$, yielding the adjacency matrix $A_{\tau }(t)$.
4. *Ricci curvature computation:* The average Ollivier–Ricci curvature $\langle \kappa_{\tau }(t)\rangle$ is computed to characterize local connectivity and network robustness.
5. *Euler characteristic computation:* The Euler characteristic is evaluated as
  $$\chi_{\tau }(t)=|V_{\tau }(t)|-|E_{\tau }(t)|,$$
  providing a global topological summary of network cohesion and fragmentation.
6. *Entropy-based descriptors:* To complement the spectral and geometric metrics, two entropy-based quantities are computed within each sliding window.

*Spectral entropy* Let $\{\lambda_{i}(t){\}}_{i=1}^{N}$ denote the eigenvalues of the correlation matrix within [t,t+τ]. A normalized spectral probability distribution is defined as


$$p_{i}(t)=\frac{\lambda_{i}(t)}{\sum_{j=1}^{N}\lambda_{j}(t)},$$

and the spectral entropy is computed as


$$H_{spec}(t)=-\sum_{i=1}^{N}p_{i}(t)\log p_{i}(t).$$

Lower values of $H_{spec}(t)$ indicate increased synchronization and reduced market complexity.

*Geometric network entropy*. Let $\{\kappa_{e}(t){\}}_{e\in E(t)}$ denote the Ollivier–Ricci curvatures associated with network edges. Following theoretical links between curvature, entropy, and robustness [13], a curvature-based probability distribution is defined as


$$q_{e}(t)=\frac{e^{-\kappa_{e}(t)}}{\sum_{e^{\prime }\in E(t)}e^{-\kappa_{e^{\prime }}(t)}},$$

and the corresponding geometric entropy is computed as


$$H_{OR}(t)=-\sum_{e\in E(t)}q_{e}(t)\log q_{e}(t).$$

This measure quantifies the heterogeneity of local geometric deformations, with lower values indicating concentrated structural fragility.

7. *Structural analysis:* The temporal evolution of $\lambda_{\max}(t)$, $\langle \kappa_{\tau }(t)\rangle$, $\chi_{\tau }(t)$, $H_{spec}(t)$, and $H_{OR}(t)$ is examined to identify regime shifts, synchronization episodes, and periods of increased systemic vulnerability.

## 4 Results and discussion

This section reports empirical evidence supporting the study’s central methodological claim: combining spectral synchronization (RMT), local geometric robustness (Ollivier–Ricci curvature), global structural cohesion (Euler characteristic), and entropy-based descriptors yields complementary information about systemic fragility that is not fully recovered by any single perspective. Results are presented at two temporal resolutions. Annual analyses provide a descriptive, coarse-grained baseline that contextualizes crisis-induced synchronization, whereas the sliding-window analysis constitutes the main empirical evidence by capturing continuous regime dynamics and event-driven structural transitions.

### 4.1 Correlation analysis

**Fig 1:**
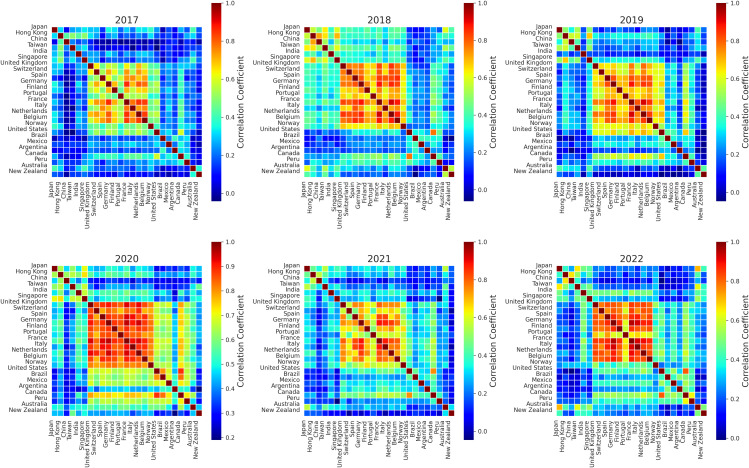
Correlation matrices from 2017 to 2022.

A year-by-year examination of global stock market correlations reveals significant structural dynamics, particularly during periods of economic disruption such as the COVID-19 pandemic (Fig 1).

Between 2017 and 2019, correlation levels remained moderate, indicating relatively stable co-movements across global markets with weaker interdependencies. In 2020, a pronounced increase in overall correlation is observed, consistent with synchronized market reactions to the economic shock induced by the COVID-19 pandemic. This phenomenon aligns with prior evidence of heightened co-movement during crisis episodes [7].

In contrast, 2021 exhibits a decline in correlations, coinciding with the onset of economic recovery and divergence of market trajectories due to country-specific policies. This pattern suggests a weakening of globally synchronized market forces, consistent with post-crisis divergence reported in the literature [19,42]. By 2022, correlation levels increase again, indicating a partial return to more coordinated market behavior, potentially influenced by global trade dynamics and macroeconomic realignments.

These observations underscore the role of large external shocks, such as COVID-19, in driving synchronized increases in global market correlation, whereas recovery phases tend to exhibit a reduction in co-movement reflecting heterogeneous economic trajectories and regional adjustments [18,19,42,43].

While these annual correlation matrices offer an interpretable overview of crisis-induced synchronization, they are inherently limited by temporal aggregation. Accordingly, this analysis serves as a descriptive baseline that motivates the subsequent dynamic investigation; the sliding-window analysis in Section 4.3 provides the main evidence on continuous regime dynamics and event-driven structural transitions.

### 4.2 A discussion of annual variations in the average Ricci curvature and Euler characteristic

**Fig 2:**
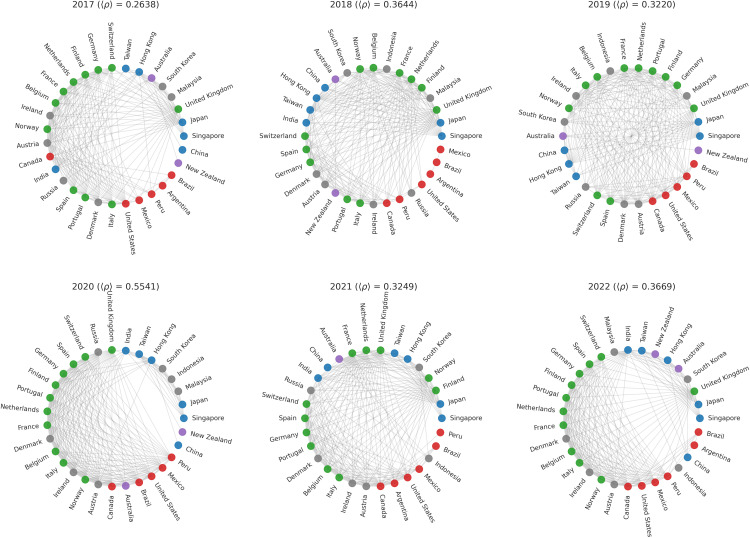
Filtered graphs for each year. Colors represent the region.

To analyze the structural evolution of the global stock market network, annual correlation matrices were used to construct yearly financial networks that reflect changes in connectivity and complexity (Fig 2). Edges were defined using a density-adaptive threshold based on the annual average correlation level. This choice intended to account for cross-year shifts in overall correlation intensity, helping to reduce mechanical network densification during crisis periods and allowing structural changes to be interpreted relative to contemporaneous market conditions.

**Fig 3:**
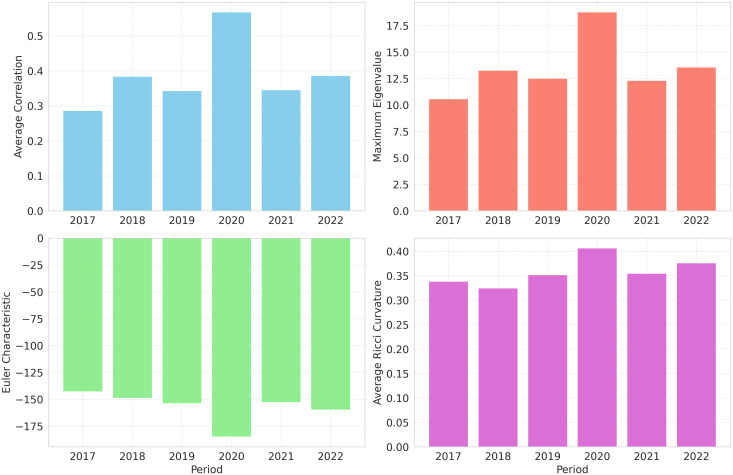
Average correlation $\langle \rho_{ij}\rangle$, maximum eigenvalue ($\lambda_{\max}$), Euler characteristic (χ), and average Ricci curvature (κ) from 2017 to 2022.

The joint assessment of the average Ricci curvature and Euler characteristic reveals substantial changes in both the geometric and topological properties of the network over 2017–2022 (Fig 3).

#### 4.2.1 Ricci curvature analysis.

The year-to-year fluctuations in average Ricci curvature provide information about network cohesion and local robustness. Higher curvature values are consistent with locally well-integrated structures, whereas lower values highlight weaker, bridge-like connections that may correspond to structural vulnerabilities or transitional phases in market interdependencies. During the pre-pandemic period (2017–2019), the average Ricci curvature remains moderate, ranging from κ=0.339 (2017) to κ=0.352 (2019) (Fig 3), suggesting that market behaviors are comparatively more regionally constrained and less globally integrated [14,15].

A marked shift occurs in 2020, when the average Ricci curvature increases to κ=0.407, coinciding with the onset of the COVID-19 crisis. This increase is consistent with heightened cohesion under crisis-induced synchronization and systemic interdependence [7,42]. In 2021, curvature decreases to κ=0.355, suggesting reduced global integration during recovery and heterogeneous policy responses [18,19,42,43]. By 2022, curvature increases again (κ=0.376), indicating a partial reconvergence of global market structure. This trend may be associated with the normalization of international trade, the stabilization of financial conditions, and the implementation of economic recovery measures, ultimately leading to renewed financial integration.

#### 4.2.2 Euler characteristic analysis.

The Euler characteristic, as a global topological invariant, complements curvature by summarizing network cohesion and fragmentation. Less negative values indicate sparser and more fragmented structures, whereas more negative values correspond to denser networks with stronger interdependencies [16,37]. Across 2017–2022, χ ranges from −143 (2017) to −185 (2020) (Fig 3). During 2017–2019, χ is relatively stable (−143 to −154), consistent with comparatively weaker global integration. In 2020, χ decreases to −185, reflecting crisis-driven densification and synchronized market behavior [7,16]. In 2021, χ increases to −160, consistent with partial loosening of connectivity during recovery [19]. In 2022, χ increases further (−149), indicating a return to moderate connectivity.

**Fig 4:**
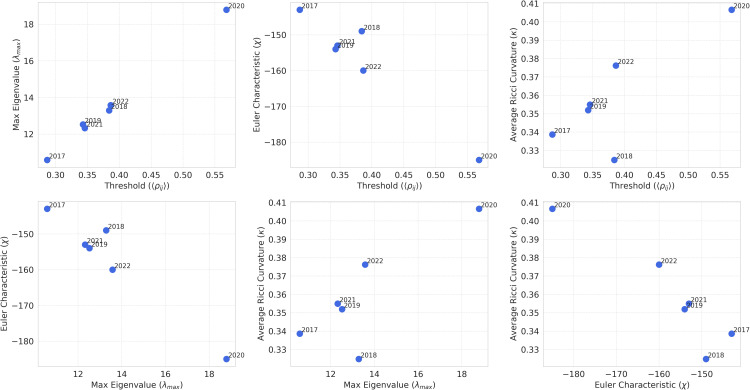
Scatter plots illustrating the relationships between topological, geometric, and spectral measures across different years.

The relationships among $\langle \rho_{ij}\rangle$, $\lambda_{\max}$, χ, and κ are summarized in Fig 4. The top row depicts associations between the average correlation and each descriptor, whereas the bottom row shows interrelations among network metrics.

Overall, the annual results indicate that 2020 consistently corresponds to the most synchronized and densified regime (high $\lambda_{\max}$, high κ, and strongly negative χ), whereas pre-pandemic years show lower cohesion and weaker interdependencies. Post-pandemic years (2021–2022) exhibit intermediate trends, consistent with partial recovery and gradual reorganization of global market structure.

#### 4.2.3 Maximum eigenvalue and collective market behavior.

The maximum eigenvalue of the correlation matrix is a standard spectral indicator of collective market behavior in RMT. Larger values indicate stronger co-movement across indices, which is often observed during crises or episodes of heightened synchronization [9,10]. As shown in Fig 3, $\lambda_{\max}$ peaks in 2020 ($\lambda_{\max}=18.79$), consistent with extreme synchronization during the COVID-19 shock. The ordering of maximum eigenvalues across years follows:


$$\lambda_{\max}^{2020}>\lambda_{\max}^{2022}>\lambda_{\max}^{2018}>\lambda_{\max}^{2019}>\lambda_{\max}^{2021}>\lambda_{\max}^{2017}.$$

This pattern supports the interpretation that major shocks induce abrupt increases in $\lambda_{\max}$ through heightened systemic interdependence.

The bottom-row scatter plots in Fig 4 show that 2020 is also characterized by the most negative Euler characteristic and the highest average curvature, consistent with a densified and cohesive network configuration. Post-pandemic years remain more interconnected than pre-pandemic years, suggesting persistent structural imprints of large disruptions.

### 4.3 Dynamic analysis: Sliding-window approach

**Fig 5:**
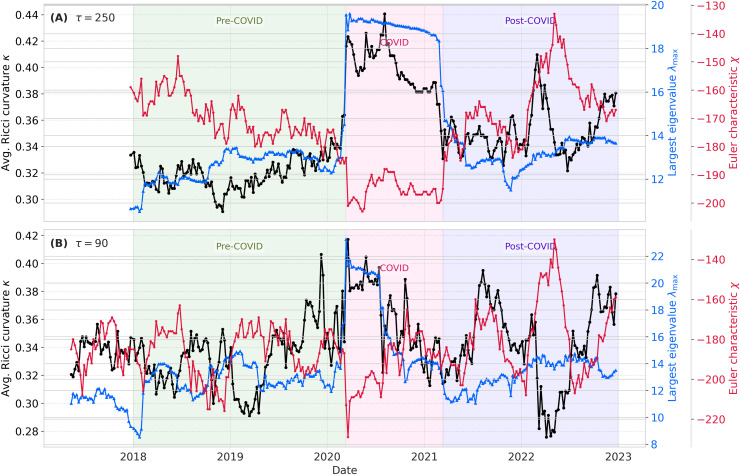
Temporal evolution of the maximum eigenvalue ($\lambda_{\max}$, blue), Euler characteristic (χ, red), and average Ricci curvature (κ, black) using sliding windows of τ=250 (A) and τ=90 (B) trading days with a shift of Δτ=5 days.

The dynamic analysis of the global stock market network (Fig 5) reveals pronounced structural transitions across the pre-COVID, COVID, and post-COVID periods. By tracking spectral, geometric, and topological descriptors within a sliding-window framework, this analysis captures the continuous evolution of market structure and highlights event-driven regime shifts that are not accessible through static annual summaries.

The maximum eigenvalue exhibits a pronounced surge at the onset of the COVID-19 pandemic, peaking in early 2020 and reflecting extreme market synchronization driven by global uncertainty. This surge is consistently detected for both window sizes, indicating that the dominant market mode is robust to the temporal resolution of the sliding-window design. Subsequently, $\lambda_{\max}$ declines as markets partially stabilize.

The Euler characteristic displays an inverse response during the crisis, becoming more negative and indicating increased network density and stronger global interconnection. This behavior is observed for both τ=250 and τ=90, although the shorter window captures sharper short-term fluctuations during acute stress episodes.

In contrast, the average Ricci curvature decreases prior to the pandemic and then increases sharply during the COVID-19 period, consistent with an event-driven reconfiguration of market connectivity. The shorter window (τ=90) yields a more temporally localized response, whereas the longer window (τ=250) emphasizes smoother regime-level trends. Post-pandemic, both $\lambda_{\max}$ and κ stabilize at levels above their pre-2020 values, suggesting persistent changes in global financial interdependencies. The persistence of this shift across temporal resolutions indicates that the post-crisis reorganization is not an artifact of window choice.

Beyond the pandemic-driven transition, the sliding-window trajectories reveal a secondary disruption during the first quarter of 2022, characterized by an increase in κ and a concurrent shift in χ. This transition temporally coincides with the onset of the Russia–Ukraine conflict (February 2022) and suggests renewed global market reconfiguration under geopolitical stress.

Given its reduced high-frequency variability and clearer separation of regime-scale dynamics, the remainder of the main dynamic analysis focuses on τ=250. Results for τ=90 are reported in the supplementary material for completeness (Fig. S1, S1 File).

To connect geometric descriptors with information-theoretic characterizations, two entropy-based measures are computed over the same sliding windows: (i) geometric network entropy $H_{OR}$, constructed from an exponential weighting of Ollivier–Ricci curvatures across edges, and (ii) spectral entropy $H_{spec}$, derived from the normalized eigenvalue distribution of the correlation matrix.

**Fig 6:**
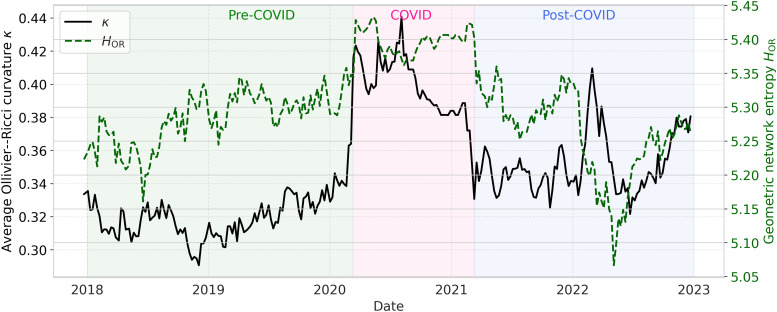
Temporal evolution of the average Ollivier–Ricci curvature κ and geometric network entropy $H_{OR}$ (τ=250 trading days).

Fig 6 shows the joint evolution of $H_{OR}$ and κ. Both measures increase markedly during the COVID-19 shock, indicating that periods of strong geometric reconfiguration are accompanied by increased heterogeneity in curvature patterns, consistent with theoretical links between curvature, entropy, and robustness [7,13,15]. In the post-COVID phase, a secondary localized deviation is observed around Q1–2022, characterized by an increase in κ together with a pronounced reduction in $H_{OR}$. This pattern indicates a renewed concentration of geometric stress on a subset of network connections and is temporally aligned with the onset of the Russia–Ukraine conflict.

The curvature–entropy association is statistically significant for τ=250 (Pearson *r* = 0.47, $p<10^{-14}$; Spearman ρ=0.39, $p<10^{-10}$), whereas the shorter window τ=90 yields weaker and noisier associations, consistent with the expected trade-off between temporal sensitivity and structural interpretability.

**Fig 7:**
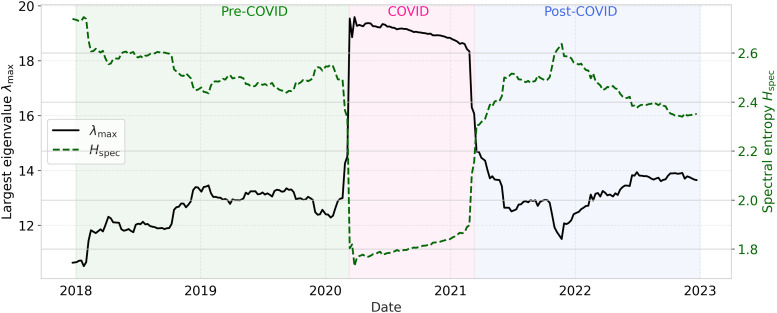
Temporal evolution of the largest eigenvalue $\lambda_{\max}$ and spectral entropy $H_{spec}$ (τ=250).

Fig 7 illustrates the inverse relationship between spectral entropy and market synchronization. During the COVID-19 crisis, $H_{spec}$ collapses while $\lambda_{\max}$ peaks, reflecting a strong concentration of variance into the dominant market mode and a reduction of market complexity. In the post-pandemic period, both quantities gradually relax toward pre-crisis levels. Around early 2022, the sliding-window trajectories reveal a secondary perturbation in the spectral descriptors, marked by a transient reconfiguration of $\lambda_{\max}$ accompanied by a corresponding adjustment in $H_{spec}$. While more moderate than the COVID-19 response, this event-driven deviation is temporally aligned with the Russia–Ukraine shock and suggests a short-lived reorganization of collective market dynamics rather than a full synchronization episode.

**Fig 8:**
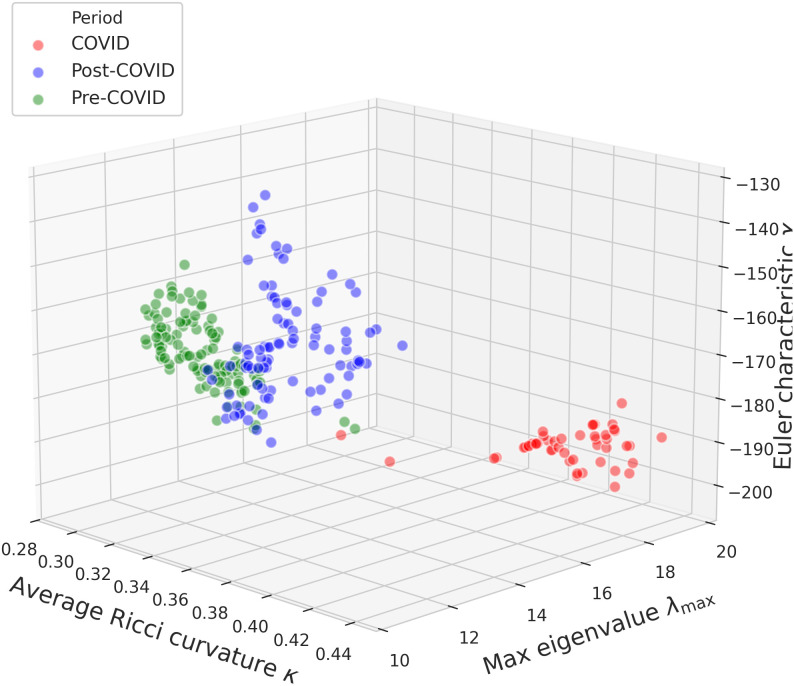
3D scatter plot. Relationship between the maximum eigenvalue ($\lambda_{\max}$), Euler characteristic (χ), and average Ricci curvature (κ) across market periods.

**Fig 9:**
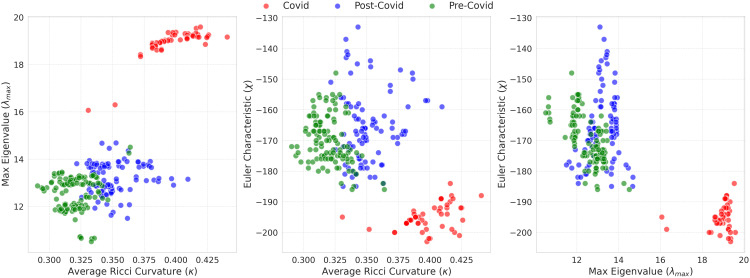
2D projections between the maximum eigenvalue ($\lambda_{\max}$), Euler characteristic (χ), and average Ricci curvature (κ).

Figs 8 and 9 further illustrate these regime changes via 3D and 2D projections, respectively, where sliding-window observations are grouped into pre-COVID, COVID, and post-COVID periods. The resulting clusters indicate that crisis episodes induce distinct and persistent structural configurations in the joint spectral–geometric–topological space.

By jointly tracking $\lambda_{\max}$, κ, χ, and entropy-based descriptors, the framework distinguishes regimes characterized by synchronization, geometric reconfiguration, and altered informational complexity. The entropy–curvature coupling further supports that systemic fragility manifests simultaneously through spectral concentration, geometric distortion, and changes in structural heterogeneity.

Finally, to assess whether the proposed descriptors provide information beyond standard network summaries, we conducted a comparative evaluation against commonly used classical metrics (average degree, density, clustering coefficient, average path length, and giant component size) computed on the same sliding-window graphs. The results indicate that the proposed measures tend to exhibit stronger regime-contrast signals during COVID-19 than several classical descriptors, while also capturing complementary geometric and topological aspects of reorganization. A detailed quantitative comparison, including normalized regime-contrast indices and effect-size estimates, is reported in supplementary information (S2 File).

### 4.4 Robustness and extension analysis

To assess whether the structural patterns identified over 2017–2022 persist beyond the original study period, we extended the sliding-window analysis to include observations from 2023 to 2024 while preserving the same methodology and parameter settings. The complete set of extended results is reported in the Supporting Information (S3 File).

**Fig 10:**
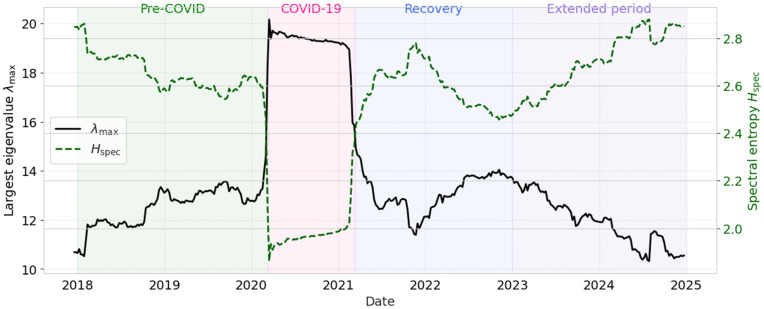
Temporal evolution of the largest eigenvalue $\lambda_{\max}$ and spectral entropy $H_{spec}$ computed using a sliding window of τ=250 trading days and a shift of Δτ=5 days over the extended period 2017–2024.

Fig 10 shows that, after the pronounced synchronization observed during the COVID-19 period, the system evolves toward a more stable, but not fully reverted, post-pandemic regime. In particular, the largest eigenvalue $\lambda_{\max}$ remains well below its 2020 peak, while the spectral entropy $H_{spec}$ exhibits a gradual recovery, indicating a reduction in extreme market-wide synchronization together with a partial restoration of structural heterogeneity.

This interpretation is further supported by the extended trajectories of the average Ollivier–Ricci curvature and the Euler characteristic reported in S3 File). Although both descriptors stabilize after 2022, they remain displaced relative to their pre-pandemic levels, suggesting that the global market network does not simply return to its original configuration. Instead, it transitions toward a reconfigured regime characterized by intermediate synchronization and persistent structural differentiation.

Taken together, these results provide temporal support for the robustness of the proposed framework and indicate that the combined spectral, geometric, and topological descriptors remain informative beyond the immediate COVID-19 shock, under the macro-financial conditions prevailing in the post-2022 period. This pattern is consistent with a reconfiguration of dependencies under the evolving post-pandemic macro-financial environment, including elevated inflation and tighter monetary conditions, as well as heightened geopolitical uncertainty since early 2022.

### 4.5 Sensitivity to network construction parameters

We further evaluated the sensitivity of the graph-based results to alternative network construction rules by comparing the baseline average-correlation threshold with fixed-density filtering (ρ=0.10,0.15,0.20), quantile-based filtering (*q* = 0.80, 0.85, 0.90), and fixed correlation thresholds ($\rho_{ij}\geq 0.30,0.40$). Full results are reported in the Supporting Information (S4 File).

As expected from the methodological design, the spectral descriptors $\lambda_{\max}$ and $H_{spec}$ are invariant across these specifications because they are computed directly from the correlation matrix rather than from the filtered graph. Accordingly, the sensitivity analysis is most informative for graph-derived quantities.

Within this setting, the average Ollivier–Ricci curvature exhibits small to moderate sensitivity to the filtering scheme ($\eta^{2}\approx 0.03$–0.05), which is consistent with its dependence on the local edge configuration induced by thresholding. Importantly, however, these differences mainly affect absolute levels rather than the overall temporal organization of the system. Across all filtering schemes, the same major regimes and transition points are recovered, including the sharp synchronization episode during COVID-19 and the subsequent stabilization phase.

Therefore, the substantive conclusions of the study do not depend on a single thresholding convention. Rather, they reflect persistent structural patterns of the financial network that remain detectable across a broad family of network construction rules.

## 5 Conclusion

The dynamic analysis of the Ricci curvature and Euler characteristic reveals clear structural changes in the global stock market network in response to external shocks. During the COVID-19 pandemic, the increase in Ricci curvature together with a marked decrease in the Euler characteristic is associated with heightened market synchronization and network densification, consistent with a period of increased systemic fragility. In the post-pandemic phase, these measures partially revert toward pre-crisis levels, although persistent structural effects remain.

The spectral analysis based on random matrix theory complements these findings. The sharp increase in the maximum eigenvalue $\lambda_{\max}$ during COVID-19 reflects extreme collective behavior, while its incomplete normalization suggests lasting changes in global market interdependence.

Beyond the pandemic, the sliding-window analysis identifies a secondary structural perturbation in early 2022, primarily captured by geometric and topological descriptors and temporally aligned with the Russia–Ukraine conflict. This result highlights the ability of these measures to detect heterogeneous, event-driven reconfigurations not fully reflected by correlation-based indicators alone.

Overall, the joint use of spectral, geometric, and topological measures—augmented by entropy-based descriptors—provides a compact multiscale representation of systemic fragility. The results indicate that these descriptors capture complementary aspects of market structure that are not fully recovered by classical network metrics, supporting their relevance for the analysis and monitoring of financial network stability.

Several limitations should be noted. The analysis relies on linear correlations and global indices; future work may incorporate nonlinear dependence measures, higher-order network representations, or asset-level data to further refine the characterization of systemic risk.

## Supporting information

S1 File Estimation and validation of embedding parameters and analysis of geometric-topological sensitivity. (DOCX)

S2 File Comparison with classical network metrics. (DOCX)

S3 File Out-of-sample validation and extended results (2023–2024). (DOCX)

S4 File Sensitivity analysis across network construction schemes. (DOCX)

## References

[1] Nikkinen J, Omran M, Sahlström P, Äijö J. Global stock market reactions to scheduled U.S. macroeconomic news announcements. Glob Financ J. 2006;17(1):92–104. doi: 10.1016/j.gfj.2006.06.003

[2] Erfanian A, Ariff M, Bhatti MI. Market tempo: Decoding information speed across global stock markets. Int Rev Econ Finance. 2024;96:103635. doi: 10.1016/j.iref.2024.103635

[3] Chuluun T. Global portfolio investment network and stock market comovement. Glob Financ J. 2017;33:51–68. doi: 10.1016/j.gfj.2016.08.002

[4] Muñoz Mendoza JA, Ferreira G, Márquez Sanders VA. Liquidity spillovers in the global stock markets: Lessons for risk management. Glob Financ J. 2023;58:100896. doi: 10.1016/j.gfj.2023.100896

[5] Jin X, Chen C, Yang X. The effect of international media news on the global stock market. Int Rev Econ Financ. 2024;89:50–69. doi: 10.1016/j.iref.2023.07.096

[6] Li Z-C, Xie C, Wang G-J, Zhu Y, Zeng Z-J, Gong J. Forecasting global stock market volatilities: A shrinkage heterogeneous autoregressive (HAR) model with a large cross-market predictor set. Int Rev Econ Financ. 2024;93:673–711. doi: 10.1016/j.iref.2024.05.008

[7] Samal A, Pharasi HK, Ramaia SJ, Kannan H, Saucan E, Jost J, et al. Network geometry and market instability. R Soc Open Sci. 2021;8(2):201734. doi: 10.1098/rsos.201734 33972862 PMC8074692

[8] Garas A, Argyrakis P, Havlin S. The structural role of weak and strong links in a financial market network. Eur Phys J B. 2008;63(2):265–71. doi: 10.1140/epjb/e2008-00237-3

[9] Laloux L, Cizeau P, Potters M, Bouchaud JP. Random Matrix Theory and Financial Correlations. Int J Theor Appl Fin. 2000;3(3):391–7. doi: 10.1142/S0219024900000255

[10] Plerou V, Gopikrishnan P, Rosenow B, Amaral LAN, Guhr T, Stanley HE. Random matrix approach to cross correlations in financial data. Phys Rev E Stat Nonlin Soft Matter Phys. 2002;65(6 Pt 2):066126. doi: 10.1103/PhysRevE.65.066126 12188802

[11] Kwapień J, Drożdż S. Physical approach to complex systems. Phys Rep. 2012;515(3–4):115–226. doi: 10.1016/j.physrep.2012.01.007

[12] Ollivier Y. Ricci curvature of Markov chains on metric spaces. J Funct Anal. 2007;256(3):810–64. doi: 10.1016/j.jfa.2008.11.001

[13] Sandhu RS, Georgiou TT, Tannenbaum AR. Ricci curvature: An economic indicator for market fragility and systemic risk. Sci Adv. 2016;2(5):e1501495. doi: 10.1126/sciadv.1501495 27386522 PMC4928924

[14] Sandhu R, Georgiou T, Reznik E, Zhu L, Kolesov I, Senbabaoglu Y, et al. Graph Curvature for Differentiating Cancer Networks. Sci Rep. 2015;5:12323. doi: 10.1038/srep12323 26169480 PMC4500997

[15] Ni C, Lin Y, Gao J, Gu X, Saucan E, Yau ST. Community detection on networks with Ricci flow. Sci Rep. 2019;9(1):9984. doi: 10.1038/s41598-019-46380-9 31477786 PMC6718387

[16] Petri G, Scolamiero M, Donato I, Vaccarino F. Topological Strata of Weighted Complex Networks. PLoS One. 2013;8(6):e66506. doi: 10.1371/journal.pone.0066506 23805226 PMC3689815

[17] Samitas A, Kampouris E, Polyzos S. Covid-19 pandemic and spillover effects in stock markets: A financial network approach. Int Rev Financ Anal. 2022;80:102005. doi: 10.1016/j.irfa.2021.102005 36536788 PMC8694798

[18] Wątorek M, Kwapień J, Drożdż S. Financial return distributions: Past, present, and COVID-19. Entropy. 2021;23(7):884. doi: 10.3390/e23070884 34356425 PMC8303836

[19] Huang W, Wang H, Wei Y, Chevallier J. Complex network analysis of global stock market co-movement during the COVID-19 pandemic based on intraday open-high-low-close data. Financ Innov. 2024;10(1). doi: 10.1186/s40854-023-00548-5

[20] Tang Y, Xiong J, Cheng Z, Zhuang Y, Li K, Xie J, et al. Looking into the Market Behaviors through the Lens of Correlations and Eigenvalues: An Investigation on the Chinese and US Markets Using RMT. Entropy (Basel). 2023;25(10):1460. doi: 10.3390/e25101460 37895581 PMC10606484

[21] Molero González L, Cerqueti R, Mattera R, Trinidad Segovia JE. The random matrix-based informative content of correlation matrices in stock markets. Chaos. 2025;35(9):093111. doi: 10.1063/5.0289031 40910858

[22] de Moraes LMT, Macêdo AMS, Vasconcelos GL, Ospina R. Eigenvalue distribution of empirical correlation matrices for multiscale complex systems and application to financial data. Phys Rev E. 2025;112(5–1):054318. doi: 10.1103/qwlr-hytm 41430879

[23] Wang X, Zhao L, Zhang N, Feng L, Lin H. Stability of China’s Stock Market: Measure and Forecast by Ricci Curvature on Network. Complexity. 2023;2023:1–12. doi: 10.1155/2023/2361405

[24] Kulkarni S, Pharasi HK, Vijayaraghavan S, Kumar S, Chakraborti A, Samal A. Investigation of Indian stock markets using topological data analysis and geometry-inspired network measures. Physica A Stat Mech Appl. 2024;643:129785. doi: 10.1016/j.physa.2024.129785

[25] Gidea M. Topological Data Analysis of Critical Transitions in Financial Networks. In: 3rd International Winter School and Conference on Network Science. Cham: Springer; 2017. p. 47–59.

[26] Akingbade SW, Gidea M, Manzi M, Nateghi V. Why topological data analysis detects financial bubbles? Commun Nonlinear Sci Numer Simul. 2024;128:107665. doi: 10.1016/j.cnsns.2023.107665

[27] Guo H, Ming Z, Xing B. Topological data analysis of Chinese stocks’ dynamic correlations under major public events. Front Phys. 2023;11. doi: 10.3389/fphy.2023.1253953

[28] de Jesus LC Jr, Fernández-Navarro F, Carbonero-Ruz M. Enhancing financial time series forecasting through topological data analysis. Neural Comput Appl. 2025;37(9):6527–45. doi: 10.1007/s00521-024-10787-x

[29] Arvanitis S, Detsis M. Mild explocivity, persistent homology and cryptocurrencies’ bubbles: An empirical exercise. AIMS Mathematics. 2024;9(1):896–917. doi: 10.3934/math.2024045

[30] Sun T. Systemic risk of systemically important financial institutions in the post‐2008 global financial crisis era: A tail risk network analysis. J Risk Insurance. 2025;92(4):950–77. doi: 10.1111/jori.70023

[31] Chen H, Wang T, Yao DD. Financial Network and Systemic Risk—A Dynamic Model. Prod Oper Manag. 2021;30(8):2441–66. doi: 10.1111/poms.13384

[32] Mantegna RN. Hierarchical structure in financial markets. Eur Phys J B. 1999;11(1):193–7. doi: 10.1007/s100510050929

[33] Qiu T, Zheng B, Chen G. Financial networks with static and dynamic thresholds. New J Phys. 2010;12(4):043057. doi: 10.1088/1367-2630/12/4/043057

[34] Nobi A, Maeng SE, Ha GG, Lee JW. Effects of global financial crisis on network structure in a local stock market. Physica A Stat Mech Appl. 2014;407:135–43. doi: 10.1016/j.physa.2014.03.083

[35] Masuda N, Boyd ZM, Garlaschelli D, Mucha PJ. Introduction to correlation networks: Interdisciplinary approaches beyond thresholding. Phys Rep. 2025;1136:1–39. doi: 10.1016/j.physrep.2025.06.002 40814354 PMC12345407

[36] Tse CK, Liu J, Lau FCM. A network perspective of the stock market. J Emp Finance. 2010;17(4):659–67. doi: 10.1016/j.jempfin.2010.04.008

[37] Ghrist R. Barcodes: The persistent topology of data. Bull Am Math Soc. 2007;45(1):61–75. doi: 10.1090/s0273-0979-07-01191-3

[38] Burda Z, Jarosz A, Nowak MA, Jurkiewicz J, Papp G, Zahed I. Applying free random variables to random matrix analysis of financial data. Part I: The Gaussian case. Quant Finance. 2011;11(7):1103–24. doi: 10.1080/14697688.2010.484025

[39] Potters M, Bouchaud JP, Laloux L. Financial applications of random matrix theory: old laces and new pieces. Acta Physica Polonica B. 2005;36(9):2767–84. doi: 10.48550/arXiv.physics/0507111

[40] Potters M, Bouchaud JP. A first course in random matrix theory: for physicists, engineers and data scientists. Cambridge University Press; 2020.

[41] Bouchaud JP, Potters M. Financial Applications of Random Matrix Theory: A Short Review. In: The Oxford Handbook of Random Matrix Theory. Oxford University Press; 2015.

[42] Wu B, Huang D, Chen M. Estimating contagion mechanism in global equity market with time‐zone effect. Financial Management. 2023;52(3):543–72. doi: 10.1111/fima.12430

[43] Ferreira E, Orbe S, Ascorbebeitia J, Álvarez Pereira B, Estrada E. Loss of structural balance in stock markets. Sci Rep. 2021;11(1):12230. doi: 10.1038/s41598-021-91266-4 34108544 PMC8190088

