# Supplementary material for: Network geometry, topology, and spectral analysis in global stock markets: Insights from using the Ricci curvature, Euler characteristic, and random matrix theory
Source: PLoS One. 2026 May 12;21(5):e0347767. doi: 10.1371/journal.pone.0347767 (PMC13166921; doi:10.1371/journal.pone.0347767)
Supplement: S1 File — (DOCX) [file pone.0347767.s001.docx]

# File S1: Sensitivity Analysis of Sliding-Window Parameters

This supplementary file reports a systematic sensitivity analysis designed to assess the robustness of the sliding-window framework adopted in the main text. The objective is to evaluate how the choice of window length τ and step size Δτ influences the stability and interpretability of the proposed spectral, geometric, and topological descriptors.

### Methodological Design

The analysis considers multiple window lengths τ ∈ {22, 60, 90, 132, 250} trading days, spanning short-term (approximately one month) to long-term (approximately one year) temporal scales. For each window length, three step sizes were examined: Δτ ∈ {1, 5, 22} trading days. This design explicitly addresses the trade-off between temporal resolution and statistical reliability.

For each (τ, Δτ) configuration, the following quantities were computed on rolling correlation networks: the maximum eigenvalue λ_max (RMT-based synchronization), the average Ollivier–Ricci curvature κ (local geometric robustness), and the Euler characteristic χ (global topological cohesion).

### Results Across Window Sizes

Figures S1 illustrate the temporal evolution of κ, χ, and λ_max for different values of τ. Across all tested window sizes, the principal qualitative signatures of the COVID-19 shock are consistently preserved: (i) a sharp increase in λ_max, indicating extreme market synchronization; (ii) a pronounced decrease in χ, reflecting network densification; and (iii) a clear regime shift in κ, signaling geometric reorganization.


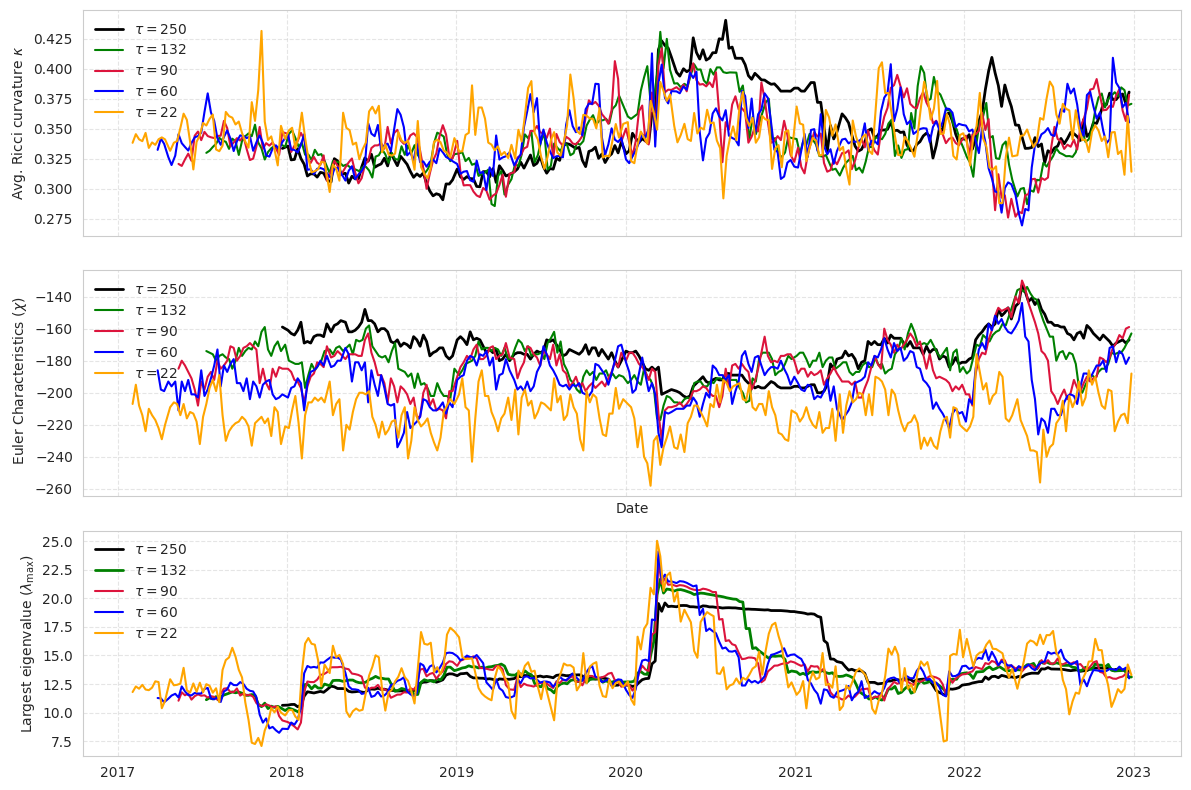


Figure S1: Comparison of Ricci curvature (κ), largest eigenvalue (λ_max) and Euler characteristic (χ) trajectories obtained for different sliding-window lengths. While shorter windows capture sharper short-term fluctuations, the relative ordering of pre-COVID, COVID, and post-COVID regimes remains stable across all tested values of τ.

Short windows (τ = 22, 60) provide high temporal sensitivity but exhibit increased high-frequency noise, which complicates the interpretation of long-term structural regimes. In contrast, longer windows (τ = 132, 250) yield smoother trajectories that more clearly separate pre-COVID, COVID, and post-COVID regimes.

### Influence of Step Size Δτ

Figure S2 compares results obtained with Δτ = 1, 5, and 22 trading days for τ = 250. While Δτ = 1 maximizes temporal granularity, it introduces redundant information and increased autocorrelation. Δτ = 22 leads to overly coarse dynamics, potentially masking abrupt transitions. The intermediate choice Δτ = 5 provides a balance between smoothness and responsiveness, capturing major systemic transitions without excessive noise.


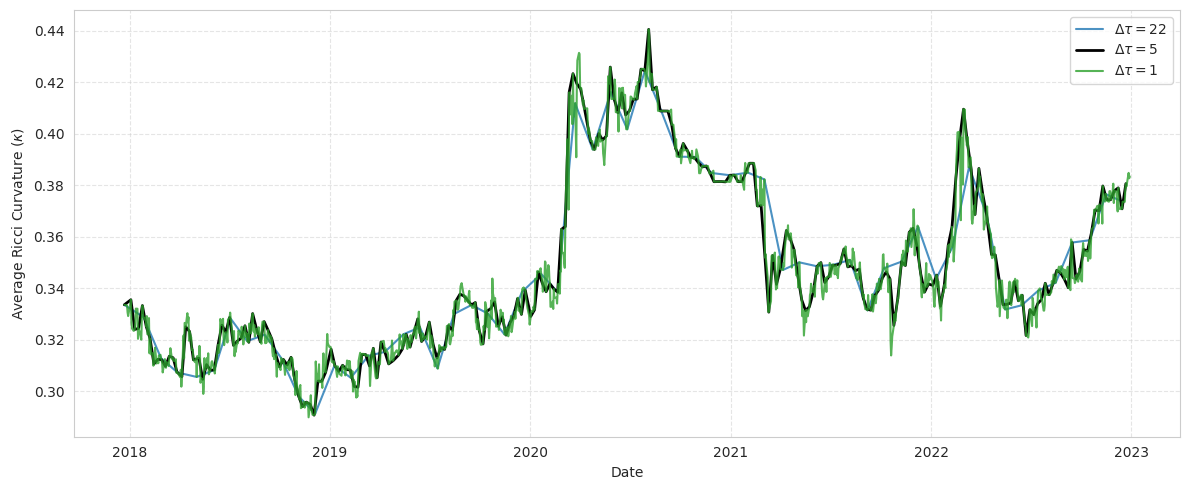


Figure S2: Average Ollivier–Ricci curvature (κ) computed using a fixed window size τ=250 trading days and step sizes Δτ={1,5,22}.

Based on this sensitivity analysis, τ=250 and Δτ=5 are adopted in the main analysis as a compromise between statistical stability, interpretability of long-term regimes, and sufficient temporal resolution to capture major systemic transitions.
