# Supplementary material for: Network geometry, topology, and spectral analysis in global stock markets: Insights from using the Ricci curvature, Euler characteristic, and random matrix theory
Source: PLoS One. 2026 May 12;21(5):e0347767. doi: 10.1371/journal.pone.0347767 (PMC13166921; doi:10.1371/journal.pone.0347767)
Supplement: S2 File — (DOCX) [file pone.0347767.s002.docx]

# File S2: Comparative Analysis with Classical Network Metrics

This supplementary file reports a comparative evaluation between the proposed spectral, geometric, and topological descriptors and commonly used classical network metrics. The analysis is conducted using sliding-window correlation networks with window size τ = 250 trading days and step size Δτ = 5 days.

## Methodology

For each sliding window, a weighted correlation network was constructed from the global stock index returns. The following families of metrics were computed:

Proposed descriptors:

- Maximum eigenvalue λ_max of the correlation matrix (Random Matrix Theory).
- Average Ollivier–Ricci curvature κ (geometric robustness).
- Euler characteristic χ (global topological invariant).

Classical descriptors:

- Average degree.
- Network density.
- Average clustering coefficient.

All metrics were z-score normalized to enable direct comparison across different scales.

## Crisis Contrast Measure

To quantify regime separation, a crisis contrast index was defined as the standardized difference between the COVID-19 period and the pre-COVID baseline. Effect sizes were quantified using Cohen’s d.

## Results

Figure S3.1 shows the temporal evolution of selected proposed and classical metrics after z-score normalization.


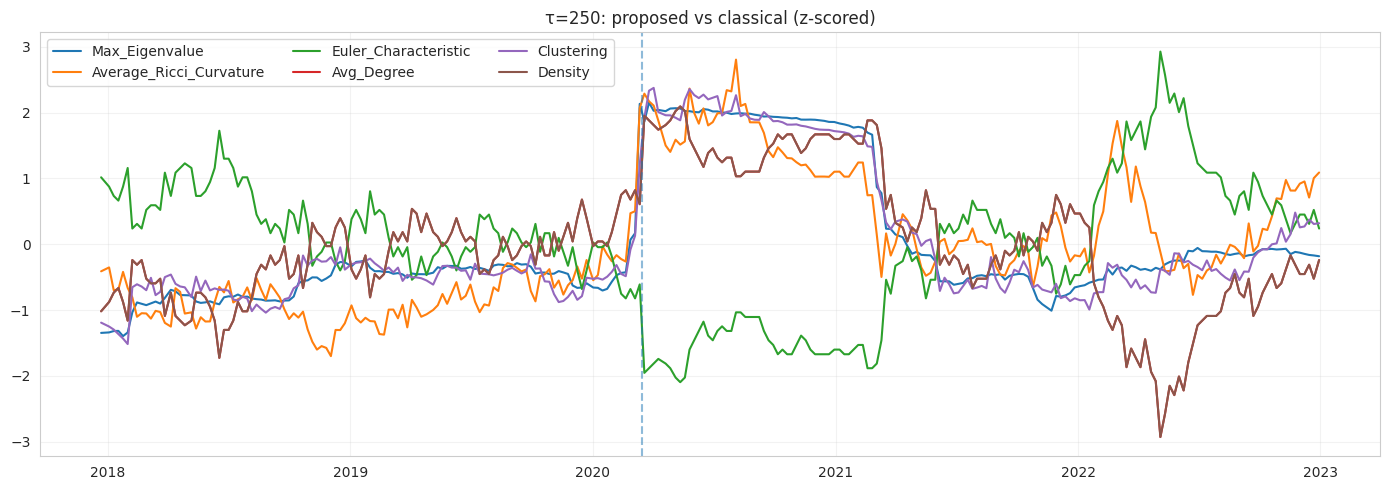


Figure S2.1: Temporal evolution of selected proposed and classical metrics

Table S2.1 summarizes the quantitative crisis contrast ranking. The proposed descriptors, particularly the maximum eigenvalue and Ricci curvature, exhibit the strongest regime contrasts, exceeding most classical metrics.

| **Metric** | **Family** | **Crisis Contrast** | **Cohen's d** | **Pre-COVID Mean** | **COVID Mean** |
| --- | --- | --- | --- | --- | --- |
| Max_Eigenvalue | Proposed | 8.36 | 5.87 | 12.52 | 18.44 |
| Average_Ricci_Curvature | Proposed | 7.04 | 5.94 | 0.32 | 0.40 |
| Euler_Characteristic | Proposed | -3.51 | -3.53 | -168.48 | -193.63 |
| Clustering | Classical | 8.13 | 5.46 | 0.42 | 0.59 |
| Avg_Degree | Classical | 3.51 | 3.53 | 12.53 | 14.10 |
| Density | Classical | 3.51 | 3.53 | 0.40 | 0.45 |

Table S2.1 Quantitative crisis contrast ranking (Covid 19).

## Discussion

The results demonstrate that the proposed descriptors capture crisis-induced structural changes more effectively than several classical network summaries. While classical metrics reflect overall densification, they provide weaker separation of post-COVID regimes and limited sensitivity to geometric heterogeneity.

In contrast, the maximum eigenvalue highlights collective synchronization, Ricci curvature captures localized geometric stress, and the Euler characteristic reflects global topological reorganization. Their combined behavior provides a multi-scale characterization of systemic fragility that is not recoverable from classical metrics alone.

## ****Event-driven robustness analysis: Russia–Ukraine shock (Q1-2022)****

### ****Methodological setup****

To complement the COVID-19 regime-contrast analysis, we performed an event-focused robustness assessment centered on the Russia–Ukraine conflict. We compared a **pre-shock baseline period** (January 2021–January 2022) against the **acute shock phase** corresponding to **Q1-2022** (February–March 2022), using the same sliding-window configuration adopted in the main analysis (τ=250. Δτ=5).

For each window, both **proposed descriptors** (maximum eigenvalue, Ricci curvature, Euler characteristic, Ollivier–Ricci entropy) and **classical network metrics** (average degree, density, clustering coefficient) were computed.

Effect sizes were quantified using **Cohen’s** d, and an absolute **crisis-contrast index** was used to rank the sensitivity of each metric to the geopolitical shock.

| **Metric** | **Family** | **Crisis Contrast** | **Cohen's d** | **Pre-** **Russia–Ukraine shock Mean** | **Russia–Ukraine shock Mean** |
| --- | --- | --- | --- | --- | --- |
| Max_Eigenvalue | Proposed | 1.67 | 1.83 | 12.39 | 13.16 |
| Average_Ricci_Curvature | Proposed | 2.33 | 2.36 | 0.35 | 0.39 |
| Euler_Characteristic | Proposed | 2.63 | 2.80 | -174.10 | -153.20 |
| Clustering | Classical | 0.63 | 0.65 | 0.76 | 0.77 |
| Avg_Degree | Classical | 3.28 | -3.39 | 15.51 | 12.21 |
| Density | Classical | 2.34 | 2.21 | 0.46 | 0.42 |

Table S2.1 Ukraine Q1-2022: pre-shock vs shock comparison.

The quantitative comparison between the pre-shock baseline and the acute Russia–Ukraine shock period reveals a consistent yet moderate structural reconfiguration of the global financial network. Unlike the COVID-19 crisis, which induced extreme synchronization and network densification, the Ukraine-related shock exhibits a more localized and heterogeneous response across spectral, geometric, and classical network descriptors.

Among the analyzed metrics, the maximum eigenvalue shows a modest increase (from 12.39 to 13.16; Crisis Contrast = 1.67), indicating a mild rise in collective market synchronization. This response is substantially weaker than the COVID-19 peak, suggesting that the geopolitical shock triggered a transient coordination of market dynamics rather than a system-wide synchronization collapse.

The average Ollivier–Ricci curvature increases from 0.35 to 0.39 (Crisis Contrast = 2.33), pointing to a strengthening of localized geometric cohesion. This rise indicates that stress becomes concentrated along specific market connections, consistent with the emergence of structurally critical transmission channels during geopolitical uncertainty.

In parallel, the Euler characteristic shifts toward less negative values (from −174.10 to −153.20; Crisis Contrast = 2.63), reflecting a partial relaxation of global network densification. Importantly, this behavior contrasts with the COVID-19 phase, where Euler characteristic decreased sharply (more negative), highlighting that the Ukraine shock induces a qualitatively different topological response: localized reorganization rather than global edge proliferation.

Classical metrics provide a complementary but less discriminative picture. Average degree and density both decrease during the shock period, confirming a reduction in overall connectivity, while the clustering coefficient shows only a marginal increase. These changes suggest that classical descriptors primarily capture connectivity loss but fail to distinguish between global sparsification and localized geometric stress.
