# Supplementary material for: Network geometry, topology, and spectral analysis in global stock markets: Insights from using the Ricci curvature, Euler characteristic, and random matrix theory
Source: PLoS One. 2026 May 12;21(5):e0347767. doi: 10.1371/journal.pone.0347767 (PMC13166921; doi:10.1371/journal.pone.0347767)
Supplement: S3 File — (DOCX) [file pone.0347767.s003.docx]

**Supporting Information**

**S3 File. Temporal extension analysis and extended results (2023–2024)**

**1. Overview**

This document presents the out-of-sample validation of the proposed framework using extended data from 2023–2024.

The analysis follows the same methodology described in the main manuscript, using sliding windows with $\tau\in\left\{ 90, 250 \right\}$ and $\Delta\tau=5$

**2. What is new**

This supplementary file extends the empirical horizon from 2022 to 2024 while preserving the same asset universe, preprocessing, rolling-window design, and descriptor definitions used in the main manuscript. Its purpose is not to introduce a new model but to assess whether the structural regimes identified over 2017–2022 remain informative in the subsequent period.

To further characterize the post-pandemic regime, Table S1 summarizes the mean values of the network descriptors across three periods (pre-COVID, COVID, and 2023–2024), together with a comparison between the extreme values observed during 2020 and the average levels in 2023–2024. The results indicate a pattern of partial normalization rather than full reversion. While the largest eigenvalue λmax​ decreases markedly from its 2020 peak (−40.9%), it remains slightly below its pre-COVID baseline, suggesting reduced systemic synchronization without complete structural recovery. In contrast, the average Ricci curvature remains close to its pre-COVID level, with only a modest deviation from the 2020 peak (−7.9%), indicating a relatively stable geometric organization. The Euler characteristic shifts toward less negative values (+19.7% relative to its 2020 trough), consistent with a relaxation of crisis-induced network densification. The spectral entropy increases substantially (+45.1%), exceeding its pre-COVID level and indicating enhanced structural heterogeneity, whereas OR entropy remains slightly below its 2020 peak (−3.6%).

| **Metric** | **Pre-COVID mean** | **COVID mean** | **Post-2022 mean** | **2020 peak/trough** | **Post-2022 minus 2020 extreme** | **% change vs 2020 extreme** |
| --- | --- | --- | --- | --- | --- | --- |
| Ricci curvature (κ) | 0.361 | 0.355 | 0.364 | 0.395 | -0.031 | -7.870 |
| Largest eigenvalue (λmax) | 12.600 | 19.225 | 11.920 | 20.173 | -8.253 | -40.911 |
| Euler characteristic (χ) | -209.904 | -222.385 | -192.663 | -240.000 | 47.337 | 19.724 |
| Olliver-Ricci entropy (H_OR) | 5.472 | 5.525 | 5.391 | 5.594 | -0.204 | -3.643 |
| Spectral entropy (H_spec) | 2.647 | 1.980 | 2.702 | 1.862 | 0.841 | 45.151 |

**Tabla S1:** Mean values of network descriptors across pre-COVID, COVID, and post-2022 periods, with comparison between 2020 extremes and 2023–2024 levels

**2. Key Findings**

The extended analysis over the period 2017–2024 reveals a clear transition toward a more stabilized regime following the COVID-19 shock, while preserving the fundamental structural patterns identified in the main analysis. As shown in Fig.1, the largest eigenvalue remains at moderate levels compared to the pronounced peak observed during the pandemic, indicating a reduction in extreme market-wide synchronization. Concurrently, both spectral entropy (Fig. 10 in the paper) and geometric network entropy (Fig.2) exhibit a gradual increase during the post-2022 period, reflecting a recovery of structural heterogeneity in the correlation network.

**
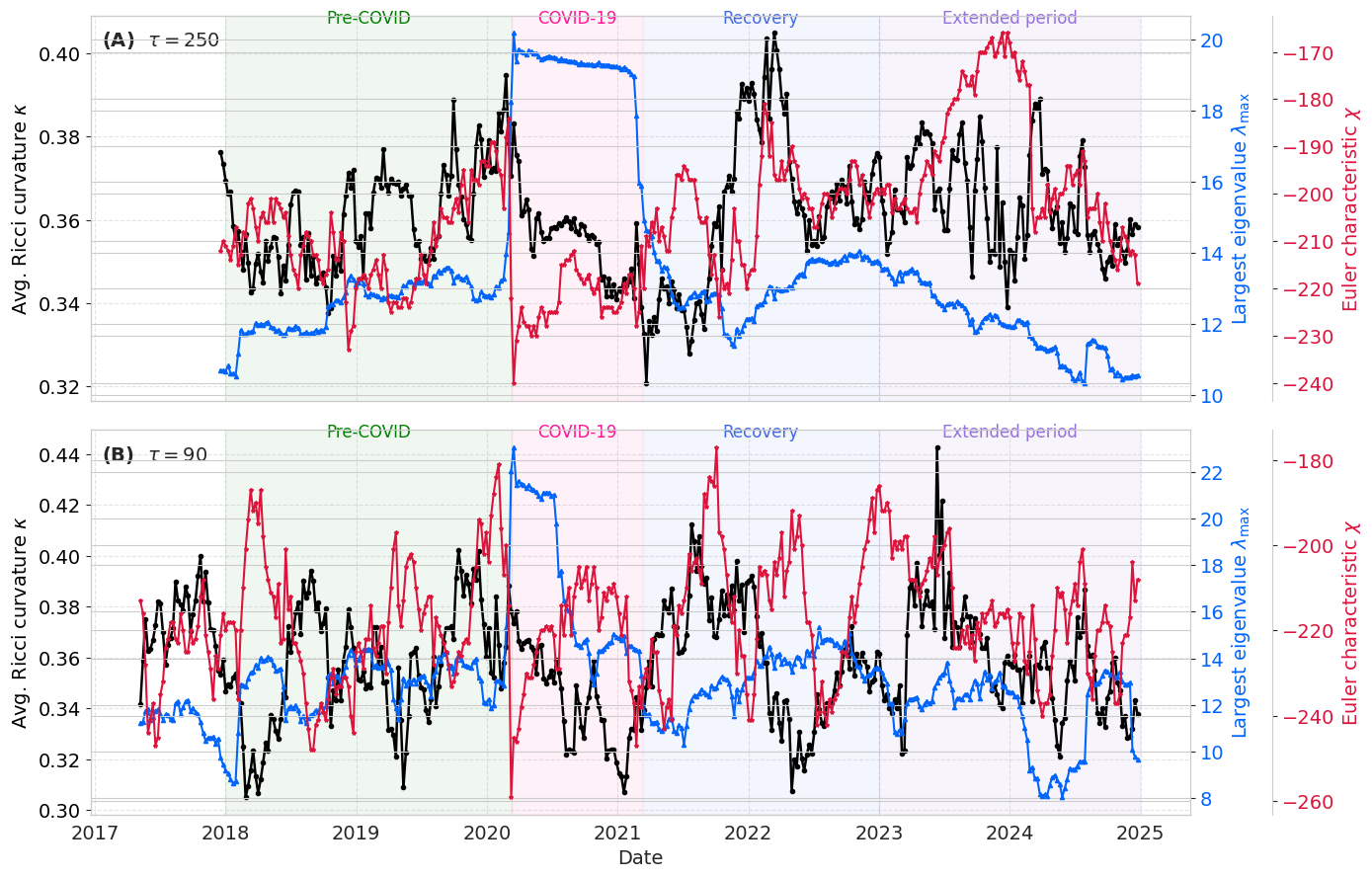
**

Fig 1: Temporal evolution of $\lambda_{max}$, average Ollivier-Ricci curvature, and Euler characteristic over 2017-2024, computed with $\tau=250$ and $\Delta\tau=5$.


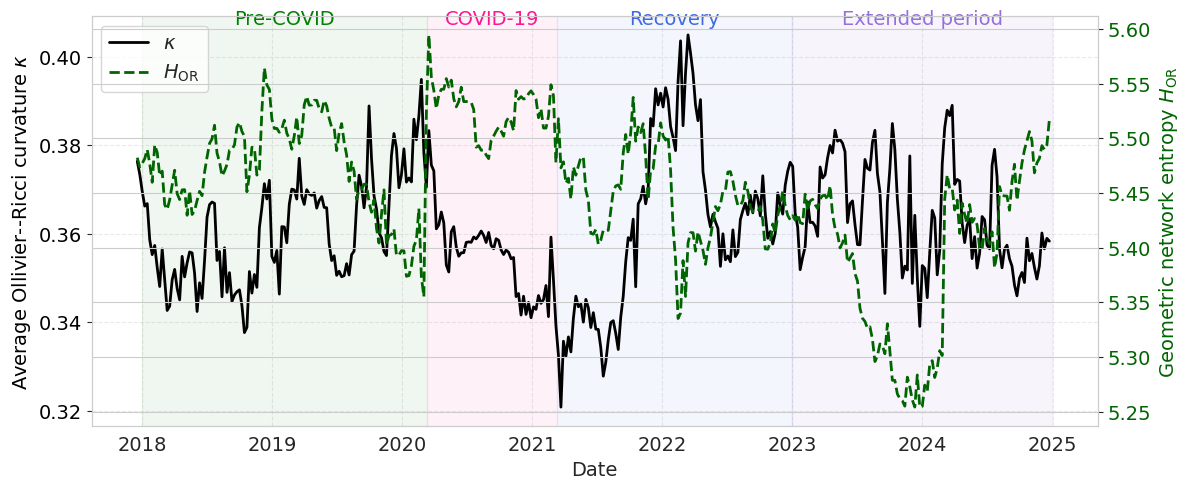


Fig 2: Evolution of average Ollivier-Ricci curvature and geometric network entropy (HOR) over 2017-2024, computed with $\tau=250$ and $\Delta\tau=5$.

From a geometric perspective, the evolution of the average Ollivier-Ricci curvature and the Euler characteristic further supports this interpretation. While both quantities display significant fluctuations during the COVID-19 phase, consistent with abrupt topological reorganization, they tend to stabilize in the extended period, albeit at levels that do not fully match pre-pandemic conditions. This suggests that the system does not revert to its original structural configuration but instead evolves toward a reconfigured regime characterized by intermediate synchronization and persistent structural differentiation.

Overall, these results indicate that the post-pandemic period is not merely a recovery phase in the traditional sense, but rather a transition toward a new equilibrium shaped by ongoing macro-financial and geopolitical dynamics, including sustained inflationary pressures, monetary policy normalization, and heightened global uncertainty. This reinforces the interpretation that the proposed geometric and spectral descriptors capture not only transient shocks but also longer-term structural adaptations in global financial networks.
