# Supplementary material for: Network geometry, topology, and spectral analysis in global stock markets: Insights from using the Ricci curvature, Euler characteristic, and random matrix theory
Source: PLoS One. 2026 May 12;21(5):e0347767. doi: 10.1371/journal.pone.0347767 (PMC13166921; doi:10.1371/journal.pone.0347767)
Supplement: S4 File — (DOCX) [file pone.0347767.s004.docx]

**Supporting Information**

**S4 File. Sensitivity analysis across network construction schemes**

**1. Overview**

This document presents a sensitivity analysis of alternative network construction schemes used to assess the stability of the graph-derived descriptors reported in the main manuscript.

We compare density‑based, quantile‑based, and fixed‑threshold filtering rules. Although the absolute levels and rank ordering of graph‑derived descriptors may vary across construction schemes, the main temporal regimes and transition points remain qualitatively stable.

**2. Methodology**

Sliding window analysis with τ = 250 and Δτ = 5 was performed under each construction rule using the same empirical framework as in the main manuscript.

**Schemes evaluated:**

- Density: 0.10, 0.15, 0.20
- Quantile: 0.80, 0.85, 0.90
- Fixed threshold: 0.30, 0.40

Because $\lambda_{max}$ and $H_{spec}$ are computed from the correlation matrix prior to graph filtering, their invariance across schemes is expected; therefore, the sensitivity analysis is primarily informative for graph-derived descriptors such as average Ricci curvature, the Euler characteristic, HOR/OR entropy, and network density.

MST-based constructions were not included because they impose a tree topology that substantially alters the interpretation of cycle‑sensitive and curvature‑based quantities, making them less directly comparable to the descriptors used in the main analysis.

**3. Results Summary**

- Spectral measures ($\lambda_{max}$, $H_{spec}$) remain invariant across all tested specifications.
- This invariance is expected, since $\lambda_{max}$ and $H_{spec}$ are computed from the correlation matrix prior to graph filtering rather tan from the filtered graph itself.
- Graph-derived descriptors, including average Ricci curvature, the Euler characteristic, HOR/OR entropy, and density, exhibit scheme-dependent shifts in their absolute magnitudes; nevertheless, the same major temporal regimes and transition points are preserved across configurations.


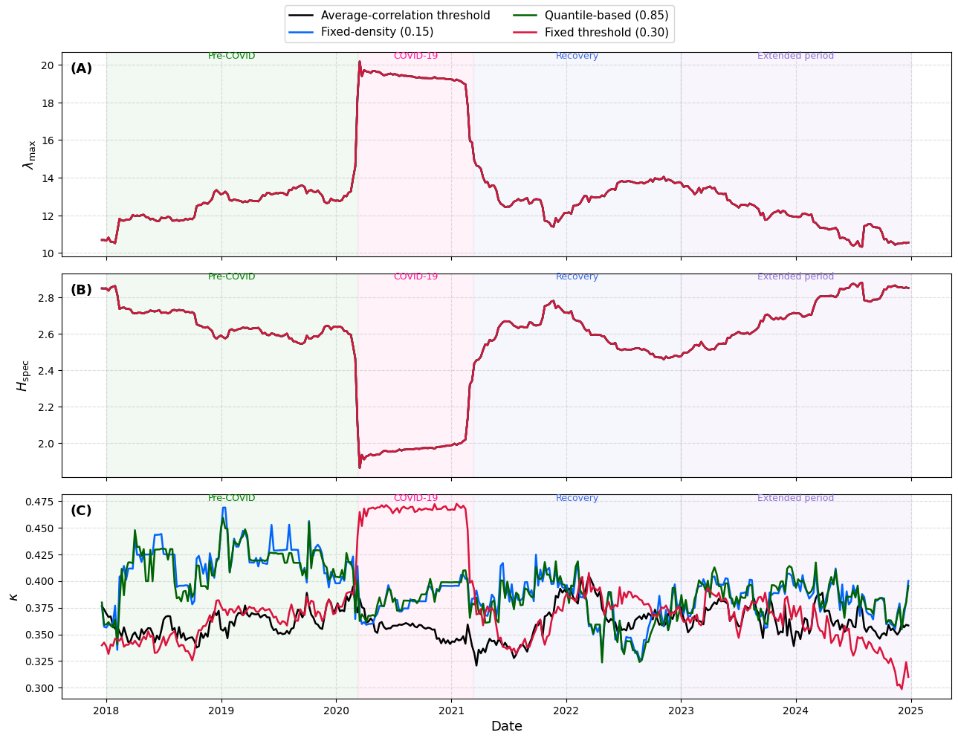


Fig. 1 Sensitivity of the selected network descriptors to alternative construction schemes and parameter choices. The panels show the temporal evolution of (A) the largest eigenvalue (B) spectral entropy and (C) average Ricci curvature under the baseline average-correlation threshold and representative alternative schemes: fixed-density filtering, quantile-based filtering, and fixed threshold filtering.

While the spectral descriptors are unchanged by construction choice, Ricci displays limited but non-negligible sensitivity in absolute magnitude. A complementary visualization of the Euler characteristic, HOR/OR entropy, and network density would further document how graph-derived descriptors shift across schemes while preserving the same crisis-related transitions.

**4. Tables**

**Table S1: Spearman correlations across schemes.** Spearman rank correlations between the baseline (average-correlation threshold) and alternative construction schemes (fixed-density, quantile-based, and fixed-threshold) are reported for each metric. For graph-derived descriptors, these correlations should be interpreted with caution: absolute levels and rank ordering may vary across schemes, even when the same temporal regimes remain qualitatively stable.

| **Reference** | **Compared_scheme** | **Parameter** | **Metric** | **Spearman** τ |
| --- | --- | --- | --- | --- |
| avgcorr | fixed_density | 0.1000 | Ricci | -0.2302 |
| avgcorr | fixed_density | 0.1500 | Ricci | -0.0489 |
| avgcorr | fixed_density | 0.2000 | Ricci | 0.2128 |
| avgcorr | quantile | 0.8000 | Ricci | 0.2058 |
| avgcorr | quantile | 0.8500 | Ricci | 0.0036 |
| avgcorr | quantile | 0.9000 | Ricci | -0.2611 |
| avgcorr | fixed_threshold | 0.3000 | Ricci | 0.3144 |
| avgcorr | fixed_threshold | 0.4000 | Ricci | 0.1629 |
| avgcorr | fixed_density | 0.1000 | *λ_max_* | 1.0000 |
| avgcorr | fixed_density | 0.1500 | *λ_max_* | 1.0000 |
| avgcorr | fixed_density | 0.2000 | *λ_max_* | 1.0000 |
| avgcorr | quantile | 0.8000 | *λ_max_* | 1.0000 |
| avgcorr | quantile | 0.8500 | *λ_max_* | 1.0000 |
| avgcorr | quantile | 0.9000 | *λ_max_* | 1.0000 |
| avgcorr | fixed_threshold | 0.3000 | *λ_max_* | 1.0000 |
| avgcorr | fixed_threshold | 0.4000 | *λ_max_* | 1.0000 |
| avgcorr | fixed_density | 0.1000 | Euler |  |
| avgcorr | fixed_density | 0.1500 | Euler |  |
| avgcorr | fixed_density | 0.2000 | Euler |  |
| avgcorr | quantile | 0.8000 | Euler |  |
| avgcorr | quantile | 0.8500 | Euler |  |
| avgcorr | quantile | 0.9000 | Euler |  |
| avgcorr | fixed_threshold | 0.3000 | Euler | 0.5320 |
| avgcorr | fixed_threshold | 0.4000 | Euler | 0.2854 |
| avgcorr | fixed_density | 0.1000 | OR_Entropy | 0.2347 |
| avgcorr | fixed_density | 0.1500 | OR_Entropy | -0.2875 |
| avgcorr | fixed_density | 0.2000 | OR_Entropy | -0.1614 |
| avgcorr | quantile | 0.8000 | OR_Entropy | -0.1659 |
| avgcorr | quantile | 0.8500 | OR_Entropy | -0.2908 |
| avgcorr | quantile | 0.9000 | OR_Entropy | 0.2012 |
| avgcorr | fixed_threshold | 0.3000 | OR_Entropy | 0.5268 |
| avgcorr | fixed_threshold | 0.4000 | OR_Entropy | 0.2644 |
| avgcorr | fixed_density | 0.1000 | Spectral_Entropy | 1.0000 |
| avgcorr | fixed_density | 0.1500 | Spectral_Entropy | 1.0000 |
| avgcorr | fixed_density | 0.2000 | Spectral_Entropy | 1.0000 |
| avgcorr | quantile | 0.8000 | Spectral_Entropy | 1.0000 |
| avgcorr | quantile | 0.8500 | Spectral_Entropy | 1.0000 |
| avgcorr | quantile | 0.9000 | Spectral_Entropy | 1.0000 |
| avgcorr | fixed_threshold | 0.3000 | Spectral_Entropy | 1.0000 |
| avgcorr | fixed_threshold | 0.4000 | Spectral_Entropy | 1.0000 |
| avgcorr | fixed_density | 0.1000 | Density |  |
| avgcorr | fixed_density | 0.1500 | Density |  |
| avgcorr | fixed_density | 0.2000 | Density |  |
| avgcorr | quantile | 0.8000 | Density |  |
| avgcorr | quantile | 0.8500 | Density |  |
| avgcorr | quantile | 0.9000 | Density |  |
| avgcorr | fixed_threshold | 0.3000 | Density | 0.5320 |
| avgcorr | fixed_threshold | 0.4000 | Density | 0.2854 |

**Table S2: ANOVA results and effect sizes.** *Analysis of variance (ANOVA) across parameter configurations for each construction scheme is summarized using the F-statistic, p-value, and effect size (η²). Small η² values indicate low sensitivity to parameter choices. To provide a complete comparison, ANOVA/effect‑size results should be reported not only for Ricci curvature,* $\lambda_{max}$*, and* $H_{spec}$*, but also for the Euler characteristic and HOR/OR entropy.*

| Scheme | Metric | *F-statistic* | *p-value* | *η² (effect size)* |
| --- | --- | --- | --- | --- |
| Fixed density | Ricci | 31.669 | 4.25 × 10⁻¹⁴ | 0.0545 |
| Fixed density | *λ_max_* | ~0 | 1.000 | 0.0000 |
| Fixed density | $H_{spec}$ | ~0 | — | 0.0000 |
| Quantile | Ricci | 30.991 | 8.07 × 10⁻¹⁴ | 0.0534 |
| Quantile | *λ_max_* | ~0 | 1.000 | 0.0000 |
| Quantile | $H_{spec}$ | ~0 | — | 0.0000 |
| Fixed threshold | Ricci | 25.811 | 4.78 × 10⁻⁷ | 0.0341 |
| Fixed threshold | *λ_max_* | ~0 | — | ~0 |
| Fixed threshold | $H_{spec}$ | 0 | 1.000 | ~0 |

***Table S3: Summary statistics by parameter configuration.*** *Mean and standard deviation of each metric across sliding windows are reported for all construction schemes and parameter values. Notation should be standardized to the symbols used in the main manuscript (*$\lambda_{max}$*,* $H_{spec}$*, HOR), decimal separators and scientific notation should be made consistent, and empty cells should be avoided unless methodologically justified.*

| **Scheme** | **Param** | **Ricci mean** | **Ricci std** | ***λ_max_* mean** | ***λ_max_***  **std** | **Euler**  **mean** | **Euler**  **std** | **OR**  **Entropy**  **mean** | **OR**  **Entropy**  **std** | **SpectralEntropymean** | **SpectralEntropystd** | **Densitymean** | **Density**  **std** |
| --- | --- | --- | --- | --- | --- | --- | --- | --- | --- | --- | --- | --- | --- |
| avgcorr |  | 0.3612 | 0.0142 | 134.71 | 25.425 | -2.030.109 | 145.919 | 54.494 | 0.0684 | 25.546 | 0.2581 | 0.4225 | 0.0260 |
| fixed_density | 0.10 | 0.3886 | 0.0393 | 134.71 | 25.425 | -220.000 | 0.00 | 40.140 | 0.0054 | 25.546 | 0.2581 | 0.0998 | 8,06E-13 |
| fixed_density | 0.15 | 0.3923 | 0.0254 | 134.71 | 25.425 | -500.000 | 0.00 | 44.154 | 0.0079 | 25.546 | 0.2581 | 0.1497 | 7,50E-13 |
| fixed_density | 0.20 | 0.3746 | 0.0288 | 134.71 | 25.425 | -780.000 | 0.00 | 47.006 | 0.0088 | 25.546 | 0.2581 | 0.1996 | 1,61E-12 |
| fixed_threshold | 0.30 | 0.3771 | 0.0405 | 134.71 | 25.425 | -2.410.545 | 1.017.048 | 55.468 | 0.3197 | 25.546 | 0.2581 | 0.4903 | 0.1813 |
| fixed_threshold | 0.40 | 0.3637 | 0.0299 | 134.71 | 25.425 | -1.514.714 | 953.875 | 51.076 | 0.4104 | 25.546 | 0.2581 | 0.3306 | 0.1700 |
| quantile | 0.80 | 0.3743 | 0.0292 | 134.71 | 25.425 | -790.000 | 0.00 | 47.094 | 0.0089 | 25.546 | 0.2581 | 0.2014 | 1,17E-12 |
| quantile | 0.85 | 0.3912 | 0.0248 | 134.71 | 25.425 | -510.000 | 0.00 | 44.271 | 0.0081 | 25.546 | 0.2581 | 0.1515 | 8,89E-13 |
| quantile | 0.90 | 0.3891 | 0.0394 | 134.71 | 25.425 | -230.000 | 0.00 | 40.317 | 0.0055 | 25.546 | 0.2581 | 0.1016 | 2,78E-14 |

Spectral metrics capture global structure and are invariant by construction. Graph‑derived metrics capture local and topological organization and therefore exhibit the expected scheme‑dependent variation in magnitude. Taken together, the results support robustness at the level of temporal regimes and structural transitions rather than strict invariance of all graph‑derived descriptors across construction choices.
